# Proteomic analysis of apoplastic fluid of *Coffea arabica* leaves highlights novel biomarkers for resistance against *Hemileia vastatrix*

**DOI:** 10.3389/fpls.2015.00478

**Published:** 2015-06-30

**Authors:** Leonor Guerra-Guimarães, Rita Tenente, Carla Pinheiro, Inês Chaves, Maria do Céu Silva, Fernando M. H. Cardoso, Sébastien Planchon, Danielle R. Barros, Jenny Renaut, Cândido P. Ricardo

**Affiliations:** ^1^Centro de Investigação das Ferrugens do Cafeeiro, Instituto de Investigação Científica TropicalOeiras, Portugal; ^2^Linking Landscape, Environment, Agriculture and Food, Instituto Superior de Agronomia, Universidade de LisboaLisboa, Portugal; ^3^Instituto de Tecnologia Química e Biológica, Universidade Nova de Lisboa (UNL)Oeiras, Portugal; ^4^Faculdade de Ciências e Tecnologia, Universidade Nova de LisboaCaparica, Portugal; ^5^Instituto de Biologia Experimental e TecnológicaOeiras, Portugal; ^6^Global Health and Tropical Medicine, Instituto de Higiene e Medicina Tropical, Universidade Nova de LisboaLisboa, Portugal; ^7^Luxembourg Institute of Science and TechnologyBelvaux, Luxembourg; ^8^Department de Fitossanidade, Universidade Federal de PelotasPelotas, Brasil

**Keywords:** coffee leaf rust (CLR), cytology, MALDI-TOF/TOF MS, 2-DE, antibody production, ELISA assay

## Abstract

A proteomic analysis of the apoplastic fluid (APF) of coffee leaves was conducted to investigate the cellular processes associated with incompatible (resistant) and compatible (susceptible) *Coffea arabica*-*Hemileia vastatrix* interactions, during the 24–96 hai period. The APF proteins were extracted by leaf vacuum infiltration and protein profiles were obtained by 2-DE. The comparative analysis of the gels revealed 210 polypeptide spots whose volume changed in abundance between samples (control, resistant and susceptible) during the 24–96 hai period. The proteins identified were involved mainly in protein degradation, cell wall metabolism and stress/defense responses, most of them being hydrolases (around 70%), particularly sugar hydrolases and peptidases/proteases. The changes in the APF proteome along the infection process revealed two distinct phases of defense responses, an initial/basal one (24–48 hai) and a late/specific one (72–96 hai). Compared to susceptibility, resistance was associated with a higher number of proteins, which was more evident in the late/specific phase. Proteins involved in the resistance response were mainly, glycohydrolases of the cell wall, serine proteases and pathogen related-like proteins (PR-proteins), suggesting that some of these proteins could be putative candidates for resistant markers of coffee to *H. vastatrix*. Antibodies were produced against chitinase, pectin methylesterase, serine carboxypeptidase, reticuline oxidase and subtilase and by an immunodetection assay it was observed an increase of these proteins in the resistant sample. With this methodology we have identified proteins that are candidate markers of resistance and that will be useful in coffee breeding programs to assist in the selection of cultivars with resistance to *H. vastatrix*.

## Introduction

Coffee leaf rust (CLR), caused by the fungus *Hemileia vastatrix* Berkeley and Broome, is the most important disease of *Coffea arabica* L. Since the first reported outbreak of CLR in 1867, that caused the eradication of coffee cultivation in Sri-Lanka, the disease has spread to all the coffee growing regions (Bettencourt and Rodrigues, 1988; Várzea and Marques, 2005). The current highly intense epidemic of CLR in Colombia and Central America has considerably affected coffee production with yield losses estimated as several 100 million dollars (Avelino et al., 2015). Although application of fungicides can provide adequate control, the use of coffee resistant varieties has been the most appropriate and sustainable strategy against this disease (Várzea and Marques, 2005).

*H. vastatrix*, like other rust fungi, is a biotrophic fungus entirely dependent on plant living cells for growth and reproduction. Rust fungi interact intimately with the plant host cells (by means of haustoria, highly specialized intracellular hyphae) modifying plant metabolism to serve the fungus nutrient needs for completion of its life cycle. This mode of interaction involves a prolonged and effective suppression of the host immune system and, at the same time, the induction of specific host genes for establishing biotrophy (Schulze-Lefert and Panstruga, 2003; Voegele and Mendgen, 2003). *H. vastatrix* starts to colonize the plant surface and after developing appressoria penetrates the host tissues through stomata, growing initially in the intercellular space before the formation of the first haustoria inside the subsidiary stomatal cells (Silva et al., 1999). The apoplast (the extracellular space that comprises cell walls and the intercellular fluid) is a metabolically very active cellular compartment, since it serves transport, environmental sensing and defense, as well as the construction and maintenance of cell walls. It is in the apoplast where the pathogen and plant first contact, and the primary defenses are activated (Agrawal et al., 2010; Floerl et al., 2012; Delanois et al., 2014; Guerra-Guimarães et al., 2014).

Plants respond to pathogen infection using a multilayer immune system, consisting of both constitutive and inducible mechanisms. The plant's ability to discriminate between its own molecules and those of the other organisms represents the first essential line of defense of any immune system (Doehlemann and Hemetsberger, 2013). The eliciting pathogen molecules (pathogen-associated molecular patterns - PAMPs) trigger in plants the first level of induced defenses or PAMP-trigger immunity (PTI). Successful pathogens deliver effectors that interfere with PTI, enabling pathogen nutrition and dispersal, and resulting in effector–triggered susceptibility (ETS). As a second defense layer, plants use resistance (R) genes to activate effector-triggered immunity (ETI) upon detection of effectors. ETI is associated with more sustained and robust immune responses including cell death by hypersensitive reaction (HR) (Jones and Dangl, 2006; Doehlemann and Hemetsberger, 2013; Delanois et al., 2014).

Coffee—*H. vastatrix* rust interactions are governed by the gene-for-gene relationship (Flor, 1942). The resistance of coffee plant is conditioned by nine major dominant genes (S_H_1–S_H_9) that have the corresponding virulence genes (*v_1_–v_9_*) in the pathogen (Rodrigues et al., 1975; Bettencourt and Rodrigues, 1988; Várzea and Marques, 2005). There is no evidence of constitutive defenses in coffee against *H. vastatrix*, but several resistance mechanisms are induced upon fungus infection (Silva et al., 2006 and references therein). Previous cytological studies have shown that for a number of coffee genotypes, the first signs of incompatibility (resistance) to *H. vastatrix* correspond to HR (Rijo et al., 1991; Silva et al., 2002, 2008). During the last decade, information on the molecular processes of the coffee-CLR interactions have been gathered using different approaches (e.g., suppression subtractive hybridization method, 454pyrosequencing and qRT-PCR) what allowed the identification of several genes putatively involved in host resistance (Fernandez et al., 2004, 2012; Ganesh et al., 2006; Diniz et al., 2012). It was thus found that more than one-quarter of the predicted proteins of the expressed sequence tags (ESTs) are disease resistance proteins, stress- and defense-proteins and components of signal transduction pathways (e.g., chitinases, beta-1,3-glucanases, PR10, lipoxygenase, AP2-type, WRKY transcription factors). Activity of oxidative enzymes (lipoxygenase, peroxidase, superoxide dismutase, and germin-like protein), phenylalanine ammonia-lyase, chitinases, and glucanases were detected in the resistance reaction. In the susceptible reaction some of these enzymes are also expressed but later (or slower) in the infection process and, therefore, are ineffective to arrest the pathogen (Maxemiuc-Naccache et al., 1992; Rojas et al., 1993; Silva et al., 2002, 2008; Guerra-Guimarães et al., 2009a,b, 2013).

Proteomics is a valuable analysis when aiming for an overview of the biochemical pathways involved in the defense response. In fact, it is an untargeted approach that provides insight into protein localization, protein-protein interactions, enzymatic complexes, or post-translational modifications (PTMs) that are essential for a better understanding of plant-pathogen interactions (Abril et al., 2011; Delanois et al., 2014; Pinheiro et al., 2014; Jorrín-Novo et al., 2015).

Based on a cytological characterized study, we conducted a 2-DE proteomic analysis of incompatible (resistant) and compatible (susceptible) *C. arabica*-*H. vastatrix* interactions with the main objectives of: further our understanding of proteins that are present in the leaf apoplast, investigate the dynamic nature of the proteins in relation to coffee-fungus interactions and link these proteins with the resistant/susceptible response pathways on the basis of their physiological role. Proteins identified by MS and that were associated with the pathogen resistance response, namely, glycohydrolases, proteases, and PR-proteins, were chosen for antibody (Ab) production. To validate these proteins as potential biomarkers of resistance, the Abs were used in an immunodetection assay. With this methodology we have identified proteins that are potential candidate markers of resistance that will be useful to assist in the selection of coffee cultivars with resistance to *H. vastatrix*.

## Materials and methods

### Biological material

Five-year-old *Coffea arabica* S4 Agaro, genotype S_H_4S_H_5, that resulted from clonally propagated stem cuttings, were grown in 50 L pots in a mixture of soil:peat:sand (1:1:1) under greenhouse conditions as previously stated (Guerra-Guimarães et al., 2014). Two races of the fungus *Hemileia vastatrix* were used in this study, one that establish a compatible interaction characterized by fungus growth and plant disease (susceptible reaction) and another one that establish an incompatible interaction characterized by a resistance response of the plant that leads to fungus death (Várzea and Marques, 2005). So, when race XV (v_4,5_) was inoculated the plant showed disease symptoms indicating it was susceptible to this fungal race and it is said that a compatible plant-fungus interaction was established. Inoculation with *H. vastatrix* race II (v_5_) showed resistant symptoms to this fungal race and it is said that an incompatible plant-fungus interaction occurred (Várzea and Marques, 2005). Fresh uredospores of *H. vastatrix* (1 mg/pair of leaves) were spread over the lower surface of young coffee leaves, as previously described (Silva et al., 2002). Healthy leaves sprayed with water and kept in the same conditions as inoculated leaves were used as control. For each coffee—rust interaction, inoculations were performed during September/October on at least three separate occasions, using different batches of spores. Leaves were collected 24, 48, 72, and 96 h after inoculation (hai) for experimental purpose.

### Light microscopy

Cross sections of infected leaf fragments made with a freezing microtome (Leica CM1850) were stained and mounted in cotton blue lactophenol to evaluate fungal growth stages (Silva et al., 2002). To detect autofluorescent cells, cross sections of infected leaf fragments were placed in 0.07 M pH 8.9 phosphate solution (K_2_HPO_4_) for 5 min, and mounted in the same solution (Silva et al., 2002). Autofluorescence and/or browning of cell contents, under blue light epifluorescence are thought to indicate plant cell death (Heath, 1998). Autofluorescence can also be used as an indicator of fungal death (Heath, 1984). Observations were made with a Leica DM-2500 microscope equipped with a mercury bulb HB 100W, blue light (excitation filter BP 450–490; barrier filter LP 515). Data were recorded from 75 to 100 infection sites/coffee-rust interaction/observation time/experiment. Since no significant differences were observed between different sets of experiments, data for each coffee-rust interaction were pooled. Arcsine-transformed percentages and Student *t*-test for statistical analysis were used.

### Plant protein extraction

The apoplastic fluid (APF) of the leaves was obtained from samples that represent a pool of 8 pairs of leaves (10 ± 2.5 g fresh weight) from 3 to 4 different plants. The leaves were vacuum infiltrated as previously described (Guerra-Guimarães et al., 2009b). Briefly, square sections of about 2 cm^2^ of leaves were vacuum infiltrated, in 100 mM Tris-HCl buffer (pH 7.6) solution, containing 50 mM L-ascorbic acid, 500 mM KCl and 25 mM 2-mercaptoethanol (at 4°C). The blotted sections were centrifuged at 5000 g, during 15 min at 4°C, and the collected APF frozen. This fraction was subsequently desalted, concentrated and purified (Guerra-Guimarães et al., 2014). APF protein quantification was made using a modified Bradford assay method (Ramagli, 1999). The purity of the APF was evaluated, prior to protein denaturing, by measuring the relative activity of malate-dehydrogenase, used as a cytosolic marker (Alves et al., 2006; Guerra-Guimarães et al., 2009b).

### 2D electrophoresis

As previously described (Guerra-Guimarães et al., 2014) IEF was performed in IPG strips with slight alterations. One hundred microgram of protein was loaded to 13 cm IPG strips (linear pH gradient of 4–7; GE Healthcare). The Ettan IPGphor (GE Healthcare) was used under the following conditions: a total of 33,000Vh at 20°C; Step-n-hold 100V-2h; Step-n-hold 30V-10h; Step-n-hold 250V-250Vh; Step-n-hold 500V-750Vh; Step-n-hold 1000V-1500Vh; Step-n-hold 2500V-2500Vh; Gradient 8000V-4h; Step-n-hold 8000V-40000Vh; maximum current setting of 50 μA per strip. After IEF, the proteins in the IPG strip were equilibrated for 15 min on a buffer (100 mM Tris–HCl pH 8.8, 6 M urea, 2% SDS, 30% glycerol, and 0.2 mg/mL bromophenol blue) containing 5 mg/mL DTT (to reduce proteins), followed by another 15 min equilibration in the same buffer but containing 25 mg/mL iodoacetamide (to alkylate proteins) instead of DTT.

The second dimension SDS-PAGE was performed at 20°C with 12% resolving gels using the Hoefer SE 600 Ruby apparatus (GE Healthcare) at 10 mA per gel, for the first 15 min, and 20 mA per gel for the next 4 h, or until the bromophenol blue dye front had run off the gel. Precision Plus Protein All Blue Standards (Bio-Rad, Hercules, CA) were used for molecular mass determinations.

### Gel staining and image analysis

For informatics analysis gels were first stained with Ruthenium II Tris (bathophenantroline disulfonate) (RuBP) according to Lamanda et al. (2004) and the images acquired in the FLA-5100 Fluorescent Image Analyzer (FujiFilm), with the LPFR filter and at 550 V and 50 μm resolution. For spot picking, the same gels were subsequently stained in Colloidal Coomassie Blue (Neuhoff et al., 1985). The image gel analysis was carried out using the Progenesis SameSpots 2D software v. 4.5 (Nonlinear Dynamics Ltd). The spot volumes were normalized using the mean value of the replicates (Grove et al., 2008) (Supplementary Table S1). One-Way ANOVA was performed between the 3 samples analyzed (resistant, susceptible, and control) using a *p*-value of 0.05. For the proteins with statistically significant changes (and a fold change >1.5) a principal component analysis (PCA) was carried out and a hierarchical clustering was performed applying a Pearson correlation using the MeV 4.9 (Supplementary Table S2).

### MS-based spot identification

Polypeptide spots (*n* = 169) whose abundance changed significantly between samples (*p*-value of 0.05 and fold change > 1.5) and were visually detected in Colloidal Coomassie Blue stained gels were excised from the gels and processed using the Tecan freedom EVO200 (Tecan, Männedorf, CH). Briefly, each sample was washed initially in a 50 mM ammonium bicarbonate solution containing 50% (v/v) methanol and dehydrated using a 75% (v/v) acetonitrile (ACN) solution and dried at 37°C. Proteins were then digested in 8 μL of trypsin Gold (Promega), 5 ng/μL trypsin in 20 mM ammonium bicarbonate. After extraction with 50% (v/v) ACN containing 0.1% (v/v) trifluoroacetic acid (TFA), the peptides were dried at 50°C and spotted on MALDI-TOF target plates. A volume of 0.7 μL of 7 mg/mL α-cyano-4-hydroxycinnamic acid in 50% (v/v) ACN containing 0.1% (v/v) TFA was added. A MALDI peptide mass spectrum was acquired using the AB Sciex 5800 TOF/TOF (AB Sciex, Foster City, CA, USA), and the 10 most abundant peaks, excluding known contaminants, were selected and fragmented.

The ProteinPilot™ software 4.0.8085 was used for database searches with an in-house MASCOT platform (version 2.3, Matrix Science, www.matrixscience.com, London, UK). All proteins were identified by search against 2 databases: an EST database of coffee containing 1527276 sequences and downloaded on September 29, 2014; a NCBInr database with the taxonomy *Viridiplantae* (http://www.ncbi.nlm.nih.gov) containing 40910947 sequences and downloaded on October 30, 2014. All searches (combined MS and 10 MS/MS spectra) were carried out using a mass window of 100 ppm for the precursor and 0.5 Da for the fragments. During the different searches the following parameters were defined: two missed cleavages, fixed carbamidomethylation of cysteine, variable oxidation of methionine or tryptophan, and tryptophan to kynurenine or double oxidation to N-formylkynurenine. The proteins identified without clear annotation have been used for BLAST analysis and the protein with the highest homology (when significant) added in Supplementary Table S3.

All identifications were manually validated and extra precursors were selected for fragmentation if the obtained data were judged as insufficient. When high quality spectra were not matched to sequences, a sequence was determined manually and in the current data set could be linked to the identified protein by allowing for more missed cleavages, semitryptic peptides, or specific modifications. Only spots considered for discussion were the ones that have an unique and significant protein identification. The spots which contained more than one protein were not considered in the study, since we don't know which protein increased/decreased.

### Further data processing

For the polypeptide spots that only gave one identified protein a subsequent bioinformatic analysis was performed. The basic information was obtained using the InterProt, UniProt, and NCBI databases. The conserved domains of each protein as well as the superfamily were determined using the NCBI tools (http://www.ncbi.nlm.nih.gov). The subcellular location assignment of the proteins were performed using TargetP 1.1, SignalP 4.1 and SecretomeP 2.0 servers (http://www.cbs.dtu.dk/services/), and the LocTree3 (https://rostlab.org/services/loctree2/) (Emanuelsson et al., 2007; Bendtsen et al., 2004; Petersen et al., 2011; Goldberg et al., 2014). The evaluation of the Transmembrane domains was carried out using Transmembrane Hidden Markov Model analysis on TMHMM server v2.0 (http://www.cbs.dtu.dk/services/TMHMM-2.0/) and the presence of a glycosylphosphatidylinositol (GPI)-anchor was carried out using GPI-anchor Predictor (http://gpcr.biocomp.unibo.it/predgpi/pred.htm) and big-PI Plant Predictor (http://mendel.imp.ac.at/gpi/plant_server.html) (Krogh et al., 2001; Eisenhaber et al., 2003; Pierleoni et al., 2008). Assignment for functional annotation of the identified proteins was based on MapMan “Bin” ontology (http://mapman.gabipd.org/web/guest/mapman) using Mercator Automated Sequence Annotation Pipeline (http://mapman.gabipd.org/web/guest/app/mercator) (Lohse et al., 2014) and Gene Ontology Annotation (GO; http://www.geneontology.org) using Blast2GO software (version 2.8.2, http://www.blast2go.de/) (Conesa and Gotz, 2008). The default parameters were used for all the programs.

### Immunodetection assays

#### Peptide selection

In order to produce antisera against the coffee apoplastic protein sequences, peptides with minimal homology (to reduce the chance of non-specific antibody binding) were selected after BLASTp search. With the overall aim to identify protein regions that are most likely accessible on its surface, the hydrophobic status was determined by the software BioEdiT. A hydrophilicity plot (calculated using the Kyte-Doolittle or the Hopp-Woods algorithm) indicates which parts of the protein are probably exposed. Structure predictions were done with Chou-Fasman plots. We selected two potential peptide candidates with typical lengths from 12 to 13 amino acids for each protein (Supplementary Table S4). Peptides were purchased from Thermo Fisher Scientific Inc. (NYSE: TMO).

#### Peptide conjugation

To increase the immunogenicity of the peptides they were carrier conjugated to ovalbumin (OVA) or bovine serum albumin (BSA). Coupling was performed using one step glutaraldehyde conjugation (Hermanson, 2013), using a 5:1 ratio peptide/protein. The BSA-peptide conjugates were used in the immunization protocol and the OVA-peptide conjugates were used in the ELISA assay.

#### Animals

CD1 male mice were obtained from the Breeding Laboratory of IHMT/UNL and were housed in cages and fed autoclaved chow and water *ad libitum*.

#### Immunization protocol

The pre-immune serum was collected by sub-mandibular bleeding and then the mice were immunized with five doses. Doses were administered via the intra-peritoneal (doses 1 and 2), intra-dermal and subcutaneous (doses 3–5) with 10 to 15 days intervals between doses. Complete Freund's adjuvant was used in dose 1 and incomplete Freund's adjuvant plus peptide adjuvant (MDP, muramyl dipeptide, 10 μg per mice) and synthetic dsRNA (Double-stranded homopolymer Poly (I:C), 10 μg per mice, Sigma-Aldrich), was used in the other doses. No adjuvant was used in the fifth dose.

#### Elisa procedure

Wells of microtiter plates (Greiner) were coated with plant extract samples (10–100 μg/ml) in 50μl of extraction buffer (0.1 M Tris-HCl, 0.5 M KCl, 0.1 mM PMSF, and 0.1% sodium sulphite, pH 7.4) or with peptides conjugated with OVA (10 μg/ml) for 1 h at 37°C. The plates were then blocked with 100 μl blocking buffer (PBS with 1% PVA, pH 7.4) for 1 h at room temperature (22°C). Polyclonal antibodies in gelatin buffer (PBS, pH 7.4, containing 0.1% gelatin) were then added at 1:500 concentration, and plates were incubated for 1 h. Secondary antibodies (anti-mouse IgM or IgG Alkaline phosphate conjugated, Sigma-Aldrich) in washing buffer [PBS, pH 7.4, containing tween 0.05% (v/v)] were added at a dilution of 1:10000 and incubated for 1 h at room temperature (22°C). The plates were incubated with chromogen/substrate [nitrophenyl phosphate (4-NPP), in 10 mM ethanolamine buffer, pH 9.6, containing 0.5 mM MgCl_2_]. The absorbance at 405 nm was checked with an ELISA microplate reader. The volume was 50 μl/well except for the blocking buffer (100 μl/well). For each antigen, the cut-off value, which differentiates positive from negative results, was set by defining the cut-off as the mean value of the normal serum group plus three standard deviations.

#### Ethics statement

Animal studies were carried out in strict accordance with the Guidelines for Proper Conduct of Animal Experiments by DGAV (Portugal) and approved (ref 0421/000/000/2013). The animal experiments were conducted in strict compliance with animal husbandry and welfare regulations. Regular veterinary care and monitoring, balanced nutrition, and environmental enrichment were provided by the IHMT-UNL.

## Results

### Fungal growth and hypersensitive host cell death

**Figure 1:**
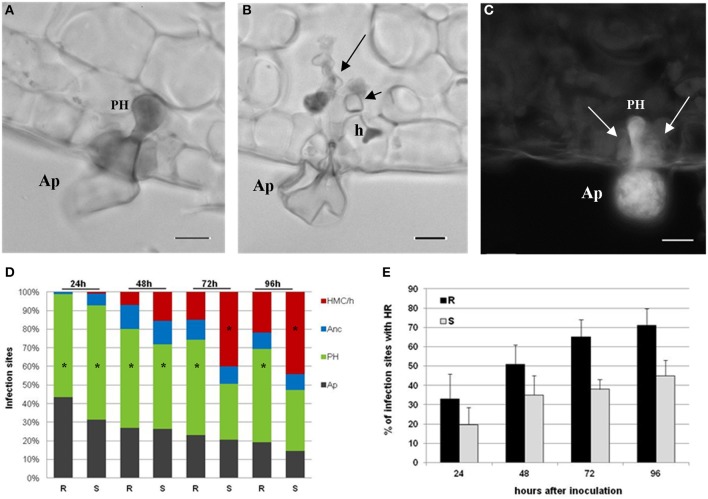
**Light micrographs of** ***H. vastatrix*** **infection sites at 72 hai**. **(A)** Appressorium (Ap) over the stomata and a penetration hypha (PH) in the resistant sample leaves, stained with cotton blue lactophenol. **(B)** Ap and intercellular hyphae (large arrow) in the susceptible sample leaves, stained with cotton blue lactophenol, being visible an haustorial mother cell (HMC) (small arrow) with a haustorium (h) in the stomatal subsidiary cell. **(C)** Autofluorescence, by blue light epifluorescence test, of guard cells (arrows) associated with a PH in the resistant sample leaves. Note that the fungal structures are also autofluorescent. (bars = 10 μm). **(D)** Percentage of infection sites with different fungal growth stages (Ap, PH, anchor - Anc, and HMC/h) in resistant (R) and susceptible (S) sample leaves at 24, 48, 72, and 96 hai. ^*^ = mode value. The weighted averages of the different fungal growth stages (Ap, PH, Anc, and HMC/h) were significantly higher in the S than in the R sample leaves at 24 hai (*t* = 4.08; *P* ≤ 0.001), 48 hai (*t* = 4.47; *P* ≤ 0.001), 72 hai (*t* = 6.77; *P* ≤ 0.001) and 96 hai (*t* = 6.91; *P* ≤ 0.001). (**E)** Percentage of infection sites with autofluorescent and/or browning cells (HR-like cell death) in R and S leaves, at 24, 48, 72, and 96 hai. The average percentages were significantly higher in the R than in the S leaves, at 24 hai (*t* = 3.71; *P* ≤ 0.001), 48 hai (*t* = 5.49; *P* ≤ 0.001), 72 hai (*t* = 12.40; *P* ≤ 0.001), and 96 hai (*t* = 8.83; *P* ≤ 0.001).

During *H. vastatrix* growth, after the differentiation of germ tubes and appressoria over stomata, the fungus infected both susceptible and resistant leaf tissues in a similar way, reaching in succession the stages of penetration hypha, anchor, and haustorial mother cell (HMC). The stomatal subsidiary cells were the first plant cells to be invaded by the haustoria. These specialized intracellular hyphae (responsible for fungus nutrients absorption) started to be formed between 24 and 48 hai. In the leaves of the resistant samples, the penetration hypha (Figure 1A) was the fungal growth stage observed with higher frequency during the all time-course of the experiment reaching about 55% at 24 hai and 50% at 96 hai, while HMC with haustorium (HMC/h) only reached 15% of infection sites at 72 hai, and did not exceed 22% at 96 hai (Figure 1D); at this stage the fungus stop growth and died. In the leaves of the susceptible samples, the penetration hypha was also the most representative stage at 24 hai (61%) and 48 hai (41%) but, later on, the HMC/h greatly increased in frequency (40% at 72 hai and 44% at 96 hai), being responsible for the successful fungal growth (Figures 1B,D). The death of the fungus was experimentally assessed by the autofluorescence of the fungal structures that, at 96 hai, reached 100% in the resistant samples and only 45% in the susceptible samples (data not shown).

The first cytological response induced by the fungus in the resistant and susceptible samples is the hypersensitive-like reaction (HR) observed initially in the stomata guard and subsidiary cells and later in mesophyll cells. At 24 hai, HR occurred for both resistant and susceptible samples reaching, respectively, 33 and 20% of infection sites, where the fungus stopped growth (at the stages of appressorium or penetration hypha). HR was always significantly higher in the resistant than in the susceptible samples at all time-points (Figures 1C,E). Only in the resistant samples was the HR observed in subsidiary stomatal cells and mesophyll cells invaded by haustoria, from 72 hai (65%) onwards (71% at 96 h).

### APF protein expression upon infection

**Figure 2:**
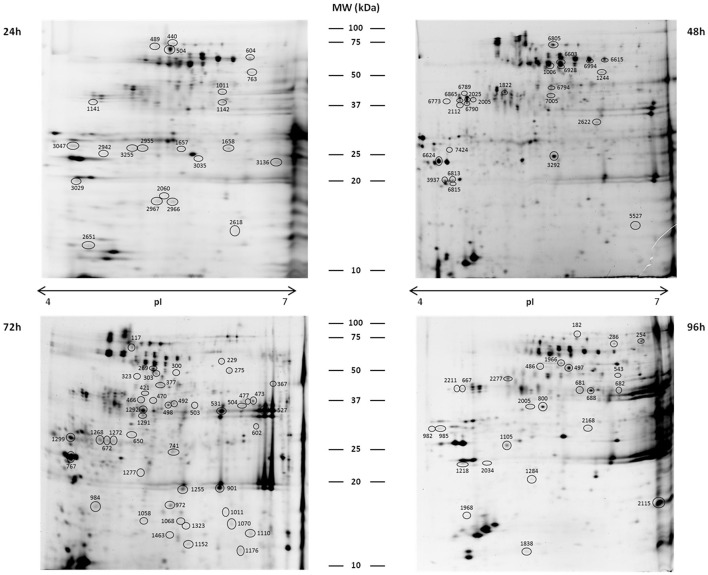
**Representative 2DE gels of coffee leaf APF proteins**. Circled spots changed significantly in abundance between samples (control, resistant, and susceptible) at 24, 48, 72, and 96 hai, and the proteins were successfully identified by MALDI-TOF/TOF-MS (see detailed information in Table 1). Gels were stained with Ruthenium II Tris.

**Figure 3:**
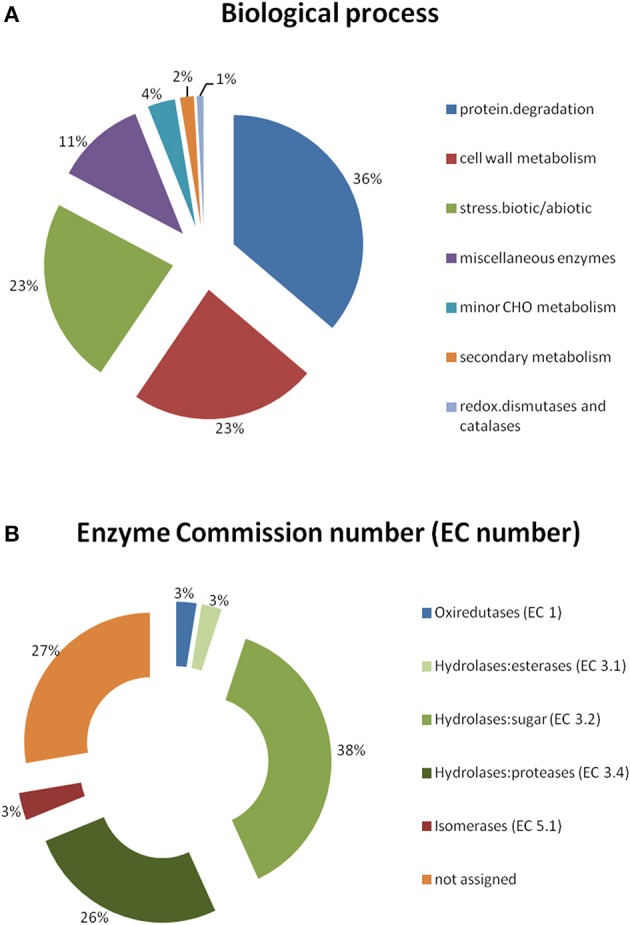
**Functional categorization of the identified coffee leaf APF proteins, based on MapMan “Bin” and GO ontology**. **(A)** Biological process; **(B)** Enzyme Commission number (EC number) of the enzymes.

The APF was obtained from resistant, susceptible and control leaves (mock-inoculated) along the *H. vastatrix* infection process (24–96 hai). Proteins were separated by 2-DE and statistical analysis of the gel patterns was performed to reveal the polypeptide spots whose volume significantly changed in abundance (*p*-value ≤ 0.05 and fold change > 1.5) between samples for each of the time-points. The number of spots that changed were 35, 37, 84, and 54, respectively, at 24, 48, 72, and 96 hai. MALDI—TOF/TOF MS analysis of the excised polypeptide spots (*n* = 169) revealed 116 spots that have only one protein identification (Figure 2, Table 1 and Supplementary Table S3). According to their conserved domains (NCBI database), these proteins belong to 23 diverse superfamilies (Table 1). Bioinformatic tools suitable for predicting secreted proteins were used in order to confirm the extracellular localization of the identified proteins. No trans-membrane domain (TMD) (TMHMM2.0) or glycosylphosphatidylinositol (GPI)-anchor (GPI-anchor Predictor and big-PI Plant Predictor) were detected in the sequences of the 116 proteins analyzed. Making use of a set of several secrete protein predictor programs (SignalP4.1, TargetP1.1, LocTree3, and SecretomeP) all the 116 proteins were indicated to be of secreted nature; from which 110 proteins had the N-terminal signal peptide typical of the classical secretory pathway (SignalP4.0 and/or TargetP1.1) and the remaining 6 proteins (that lack the classical terminal signal peptide) were recognized as leaderless secretory proteins (SecretomeP program) (Supplementary Table S5). Overall, the results confirm the high quality of the APF samples, since no or little cytoplasmic contamination was detected (APF activity of malate dehydrogenase was always less than 5% of the activity of total leaf homogenates). The functional categorization of the identified proteins was performed, gathering information from different annotation tools (GO ontology and MapMan “Bin”). Annotation revealed that the identified proteins were involved in: protein degradation (36%), cell wall metabolism (23%), stress/defense (23%), miscellaneous enzyme families (11%), minor carbohydrates (CHO) metabolism (4%), secondary metabolism (2%), and redox (1%) (Table 1 and Figure 3A). Seventy three percent of these proteins have EC numbers (Blast2GO analysis) which mainly represent hydrolases, particularly sugar hydrolases and peptidases/proteases (Figure 3B). Analyzing the changes along the infection process it is more evident that the % of proteins involved in proteolysis decreased after an initial increase (53% at 24 hai and 12% at 96 hai) while the % of proteins involved in stress/defense increased along the infection process (9% at 24 hai and 40% at 96 hai). A few proteins are present at all time-points, namely, xylosidases, mannosidases, chitinases, subtilases, and aspartic proteases.

**Table 1:** **Annotation of the coffee leaf apoplastic proteins that changed in abundance along the infection process**.

**Biological process ^a^**	**Spot no.^b^**	**Protein identity [species]^c^**	**GI acc. no.^d^**	**Superfamily^e^**	**hai^f^**	**Change relatively to control^g^**
Cell wall.biosynthesis	498	beta-D-galactosidase [*Pyrus pyrifolia*]	61162203	GH35	72	R ↓
	492	PREDICTED: beta-galactosidase 8-like isoform 1 [*Glycine max*]	356543464	GH35	72	R ↓
Cell wall.degradation	604	beta-D-xylosidase 1 precursor [*Solanum lycopersicum*]	350534908	GH3	24	R and S↓
	3035	beta-D-xylosidase 1 precursor [*Solanum lycopersicum*]	350534908	GH3	24	S↑
	1006, 1822, 6603, 6615, 6794, 6928, 6994, 7005	beta-D-xylosidase 1 precursor [*Solanum lycopersicum*]	350534908	GH3	48	R and S↓
	269, 323, 377, 421, 1070, 1110, 1463	PREDICTED: beta-xylosidase/alpha-L-arabinofuranosidase 2-like [*Nicotiana sylvestris*]	698512394	GH3	72	R↑
	1105	Lysosomal beta glucosidase [*Morus notabilis*]	587840624	GH3	96	R ↓
	543	PREDICTED: beta-xylosidase/alpha-L-arabinofuranosidase 2-like [*Nicotiana sylvestris*]	698512394	GH3	96	S↓
	486	Alpha-L-fucosidase 1 [*Theobroma cacao*]	590672071	Alpha-amylase	96	R↑
	1966	*Alpha-L-fucosidase [Medicago truncatula]*	357444199	Alpha-amylase	96	R↑
	497	PREDICTED: alpha-L-fucosidase 1-like [*Vitis vinifera*]	359490232	Alpha-amylase	96	R↑
Cell wall	367	pectin methylesterase [*Coffea arabica*]	384597517	Pectinesterase	72	R ↑
	1068	PREDICTED: pectinesterase-like [*Nicotiana sylvestris*]	698444078	Pectinesterase	72	R ↑
	1838	PREDICTED: L-ascorbate oxidase homolog [*Brassica rapa*]	685338488	Cupredoxin	96	S ↑
Minor CHO metabolism	972	PREDICTED: aldose 1-epimerase-like isoform X8 [*Nicotiana sylvestris*]	698588431	Aldose_epim	72	R ↓
	1152, 1323	PREDICTED: aldose 1-epimerase-like [*Citrus sinensis*]	568858025	Aldose_epim	72	R and S↓
	2168	non-cell-autonomous protein pathway1 [*Nicotiana tabacum*]	15824565	Aldose_epim	96	R ↑
Miscellaneous enzymes. acid and other phosphatases	1658	calcineurin-like phosphoesterase [*Manihot esculenta*]	496474724	Metallophosphatases	24	R ↑
	741	PREDICTED: purple acid phosphatase 15 [*Nicotiana sylvestris*]	698476727	Metallophosphatases	72	R ↑
Miscellaneous enzymes. GDSL-motif lipase	3136	GDSL-motif lipase/hydrolase family protein [*Populus trichocarpa*]	566199057	SGNH_hydrolase	24	R and S↓
Miscellaneous enzymes.	1142	beta-galactosidase [*Camellia sinensis*]	575456452	GH35	24	R and S↓
gluco-, galacto- and	763	alpha-mannosidase precursor [*Solanum lycopersicum*]	350538359	GH38	24	R and S↓
mannosidases.alpha-galactosidase	1244	PREDICTED: lysosomal alpha-mannosidase-like [*Nicotiana sylvestris*]	698450172	GH38	48	R and S↓
	300, 503	PREDICTED: lysosomal alpha-mannosidase-like [*Nicotiana sylvestris*]	698450172	GH38	72	R ↓
	254, 286	PREDICTED: lysosomal alpha-mannosidase-like [*Glycine max*]	356508869	GH38	96	R ↑
	681, 688	beta-galactosidase [*Camellia sinensis*]	575456452	GH35	96	S↑
	182	Alpha-xylosidase 1 [*Theobroma cacao*]	590700766	GH31	96	R ↑
Protein.degradation. aspartate protease	1141	PREDICTED: protein ASPARTIC PROTEASE IN GUARD CELL 1-like [*Solanum tuberosum*]	565349288	pepsin_retropepsin	24	R and S↓
	1657	unnamed protein product [*Coffea canephora*]	661898488	pepsin_retropepsin	24	S↑
	6773, 7424	PREDICTED: protein ASPARTIC PROTEASE IN GUARD CELL 1-like [*Solanum tuberosum*]	565349288	pepsin_retropepsin	48	S↑
	2005, 2025, 2112, 6789, 6790, 6865	PREDICTED: protein ASPARTIC PROTEASE IN GUARD CELL 1-like [*Solanum tuberosum*]	565349288	pepsin_retropepsin	48	R and S↓
	275	PREDICTED: basic 7S globulin [*Vitis vinifera*]	225436984	pepsin_retropepsin	72	R ↑
	504	PREDICTED: aspartic proteinase nepenthesin-1-like [*Nelumbo nucifera*]	720054046	pepsin_retropepsin	72	R ↑
	527	PREDICTED: aspartic proteinase nepenthesin-1-like [*Solanum tuberosum*]	565341835	pepsin_retropepsin	72	R ↑
	667, 2211	PREDICTED: protein ASPARTIC PROTEASE IN GUARD CELL 1-like [*Solanum tuberosum*]	565349288	pepsin_retropepsin	96	R ↑
Protein.degradation. cysteine protease	2942	cysteine proteinase aleuran type [*Nicotiana benthamiana*]	71482942	Peptidase_C1	24	R ↑
Protein.degradation. serine protease	466	serine carboxypeptidase, putative [*Ricinus communis*]	255553418	Peptidase_S10	72	R ↑
	650, 984	PREDICTED: serine carboxypeptidase-like 40-like [*Citrus sinensis*]	568858842	Peptidase_S10	72	R ↑
	1268	PREDICTED: serine carboxypeptidase-like 40 isoform X1 [*Vitis vinifera*]	225449979	Peptidase_S10	72	R ↑
Protein.degradation. Subtilases	489	subtilisin-like protease preproenzyme [*Nicotiana tabacum*]	253740260	Peptidases_S8_S53	24	R and S ↑
	504	subtilisin-like protease preproenzyme [*Nicotiana tabacum*]	253740260	Peptidase_S8_S53	24	R and S↓
	1011	PREDICTED: subtilisin-like protease [*Nicotiana tomentosiformis*]	697119321	Peptidases_S8_S53	24	R and S↓
	2060, 2966, 2967	PREDICTED: subtilisin-like protease [*Nicotiana tomentosiformis*]	697119321	Peptidases_S8_S53	24	R ↑
	3255	PREDICTED: subtilisin-like protease [*Solanum lycopersicum*]	723696627	Peptidases_S8_S53	24	R and S ↑
	440	PREDICTED: subtilisin-like protease [*Solanum lycopersicum*]	723695307	Peptidases_S8_S53	24	R and S ↑
	2622, 6805	PREDICTED: subtilisin-like protease [*Vitis vinifera*]	225458653	Peptidases_S8_S53	48	R and S ↓
	473	PREDICTED: subtilisin-like protease [*Nicotiana tomentosiformis*]	697119321	Peptidases_S8_S53	72	R ↓
	477	PREDICTED: subtilisin-like protease [*Nicotiana tomentosiformis*]	697119321	Peptidases_S8_S53	72	R and S ↑
	470, 602, 672, 1272, 1291, 1292	PREDICTED: subtilisin-like protease [*Nicotiana tomentosiformis*]	697119321	Peptidases_S8_S53	72	R ↑
	1011	PREDICTED: subtilisin-like protease [Nicotiana sylvestris]	698522359	Peptidases_S8_S53	72	R ↑
	117, 303	PREDICTED: subtilisin-like protease [*Nicotiana sylvestris*]	698557660	Peptidases_S8_S53	72	S ↓
	2277	subtilisin-like protease preproenzyme [*Nicotiana tabacum*]	253740260	Peptidases_S8_S53	96	R ↓
Redox	5527	copper-zinc superoxide dismutase 4, partial [*Diospyros oleifera*]	383386153	Cu-Zn_SOD	48	S↑
Secondary metabolism	2618	berberine bridge enzyme [*Hevea brasiliensis*]	341819340	FAD_binding	24	R ↑
	1176	PREDICTED: reticuline oxidase-like protein [*Prunus mume*]	645238406	FAD_binding	72	R ↑
Stress/Defense	3029	osmotin [*Piper colubrinum*]	161375756	GH64-Thaumatin-like	24	R ↑
	2651	germin-like protein, partial [*Genlisea aurea*]	527204558	Cupin	24	S ↑
	2955	PREDICTED: cysteine-rich repeat secretory protein 55-like [*Citrus sinensis*]	568862722	Stress-antifungal	24	R ↑
	3047	chitinase 1 [*Theobroma cacao*]	590589913	GH18_chitinase-like	24	R and S ↑
	6624	chitinase family protein [*Populus trichocarpa*]	566206109	GH18_chitinase-like	48	S ↓
	3937, 6813, 6815	chitinase family protein [*Populus trichocarpa*]	566206109	GH18_chitinase-like	48	R and S ↓
	3292	chitinase family protein [*Populus trichocarpa*]	566253335	GH18_chitinase-like	48	R and S ↓
	767	chitinase family protein [*Populus trichocarpa*]	550305695	GH18_chitinase-like	72	S ↓
	1277	germin-like protein 10 [Arabidopsis thaliana]	42572763	Cupin	72	S ↑
	1058	germin-like protein [*Camellia sinensis*]	344221931	Cupin	72	R ↓
	229, 531, 901, 1255	germin-like protein, partial [*Genlisea aurea*]	527204558	Cupin	72	R ↓
	1299	PREDICTED: pathogenesis-related protein 5-like [*Nicotiana sylvestris*]	698527087	GH64-Thaumatin-like	72	R ↑
	1284	putative NtPRp27-like protein [*Atropa belladonna*]	14329814	GluZincin	96	S↑
	982	chitinase 1 [*Theobroma cacao*]	590589913	GH18_chitinase-like	96	S↑
	985	PREDICTED: chitinase 2-like [*Prunus mume*]	645217067	GH18_chitinase-like	96	S↑
	1968	pathogenesis-related 1 protein [*Coffea canephora*]	485993076	SCP_PR-1_like	96	S ↑
	2115	pathogenesis-related 1 protein [*Coffea canephora*]	485993076	SCP_PR-1_like	96	R ↓
	682	germin-like protein, partial [*Genlisea aurea*]	527204558	Cupin	96	R ↑
	1218	thaumatin-like protein [*Actinidia deliciosa*]	190358875	GH64-Thaumatin-like	96	R and S ↑
	800, 2005	beta-1,3-glucanase, basic [*Coffea arabica x Coffea canephora*]	37223498	GH17	96	R and S ↑
	2034	osmotin [*Piper colubrinum*]	161375756	GH64-Thaumatin-like	96	R and S ↑

a Functional characterization of the proteins based on MapMan “Bin” and GO ontology.

b The number that identified protein spots on 2-D apoplastic gel.

c The peptide identification based on homology to proteins characterized in different species by BLASTp. search on NCBI Viridiplantae and ESTcoffee databases.

d The accession number from GenBank assigned to the polypeptide after MS/MS analysis.

e Superfamily according to NCBI classification. GH, Glycoside Hydrolase; SGNH_hydrolase, diverse family of lipases and esterases; FAD_binding, flavodoxin binding oxiredutase; GluZincin, thermolysin-like peptidases including several zinc-dependent metallopeptidases.

f hours after inoculation with H. vastatrix.

g Samples R (resistant), S (susceptible) that change in abundance relatively to control, ↑ (increase), ↓ (decrease).

### APF proteins associated with resistance and susceptibility

**Figure 4:**
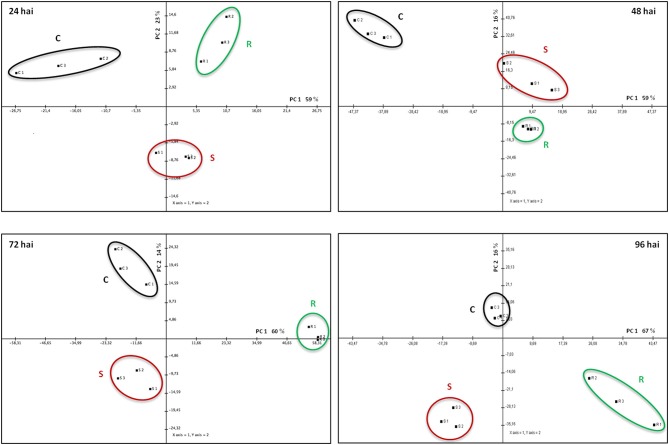
**Principal Component Analysis (PCA) performed for the spots whose volume significantly changed in abundance (*****p*****-value < 0.05), for each time-point of the infection (24–96 hai)**. Distinct groups were obtained per sample: control (C), resistant (R), and susceptible (S).

**Figure 5:**
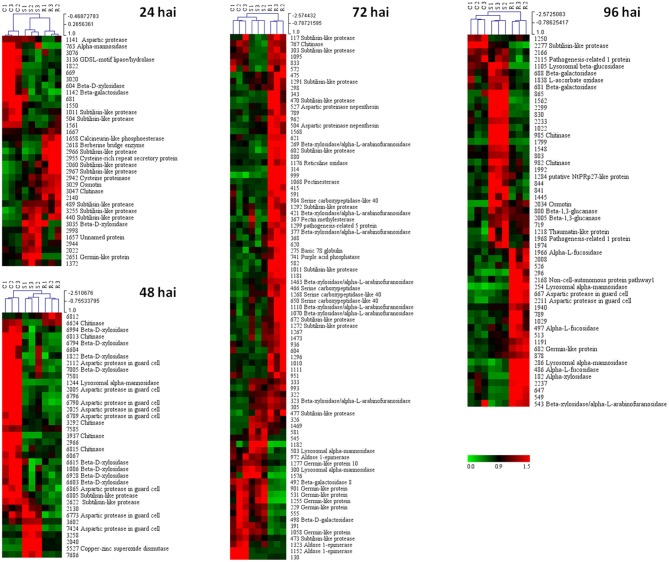
**Hierarchical cluster analysis of the proteins that significantly changed in abundance (*****p*****-value < 0.05) between control (C), resistant (R), and susceptible (S) samples, for each time-point of the infection (24–96 hai)**. The signals are shown in a red-green color scale, from a gradient of red (higher expression) to green (lower expression).

A Principal Component Analysis (PCA) was performed for the spots whose volume significantly changed in abundance during the infection. This analysis revealed a clear separation of the three samples (resistant, susceptible, and control) for each of the four time-points, the two first axes always representing more than 70% of the total variance (Figure 4 and Supplementary Table S2). To visualize the relative accumulation of the spots in the resistant (R) and the susceptible (S) samples, a hierarchical cluster analysis was performed (Figure 5). At 24 hai, the protein patterns for the two infected samples showed differences mainly concerning an increase in proteolysis and in stress/defense in the R samples (e.g., cysteine proteinases, subtilases, berberine bridge enzyme, cysteine-rich repeat secretory protein, osmotin, and chitinase). Changes in a calcineurin-like phosphoesterase and a GDSL-motif lipase/hydrolase (miscellaneous enzyme families) are also of significance. At 48 hai, it is remarkable that the two infected samples did not markedly differ from each other, both showing a strong decrease in abundance for the same proteins, e.g., beta-D-xylosidase, chitinases, and aspartic proteases. It is at 72 hai, that the main differences between R and S samples started to be evident. Most of the proteins that increase in the R sample at 72 hai are involved in proteolysis (e.g., subtilases and serine carboxypeptidases) and in cell wall degradation/modification (e.g., beta-xylosidase/alpha-arabinofuranosidases, chitinase, glucanase and pectin methylesterase, purple acid phosphatase, reticuline oxidase). However, a strong increase in stress/defense proteins (e.g., PR-1, osmotin, chitinases, thaumatin-like, NtPRp27 protein, and beta-1,3-glucanase) and beta-galactosidases were observed mostly in the S sample, at 96 hai. There is a noticeable increase in alpha-L-fucosidase proteins in the R sample at 96 hai.

### Immunodetection assay

**Figure 6:**
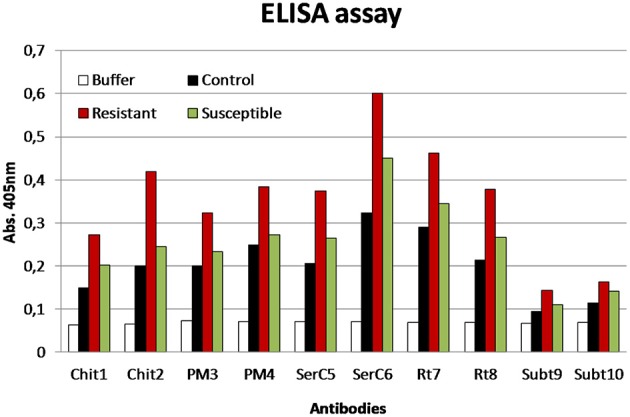
**ELISA assay using the antibodies produced against different proteins: chitinases (Chit), pectin methylesterase (PM), serine carboxypeptidase (SerC), reticuline oxidase (Rt), and subtilases (Subt)**. Antigen samples were control, resistant, and susceptible coffee leaf extracts with 72 hai with *H. vastatrix* (100 μg/ml).

Some of the identified proteins, referred above, were selected as antigen for the production of antibodies, such as, chitinase, pectin methylesterase, serine carboxypeptidase, reticuline oxidase, and subtilase. Peptides corresponding to these proteins were synthesized and after conjugation with BSA and OVA allowed the production of specific antibodies. The results obtained show a higher level of detection of those proteins in the R than in the S or control samples (Figure 6).

## Discussion

We have been studying the APF coffee leaf proteins in response to *H. vastatrix* infection (Guerra-Guimarães et al., 2009b, 2013), and recently, we have characterized the proteome of APF healthy coffee leaves (Guerra-Guimarães et al., 2014). With the present study we complement the knowledge on the importance of the proteins present in this sub-cellular compartment, particularly in relation to pathogen defense. In addition to the proteins previously found, a further seven protein superfamilies were now identified in the APF of coffee leaves (control sample), making a total of 29 protein superfamilies. The new identified protein superfamilies are mainly PR proteins, phosphatases and oxi-reductases, highlighting the existence of an important constitutive defense mechanism in *C. arabica* leaves against pathogens. We further studied the *C. arabica-H. vastatrix* pathosystem aiming to discover changes in the leaf APF proteome during the evolution of the infection process (24 hai-96 hai) in both incompatible and compatible interactions (R and S samples). The results obtained support the existence of two phases of defense responses, an initial/basal response and a later/specific response. The number of proteins involved in the initial/basal phase (24–48 hai), is half of the number involved in the late/specific phase (72–96 hai), grouped in 23 protein superfamilies of which four are present only in the initial phase, nine in the late phase and the remaining 10 in both phases.

### Initial/basal defense responses

The identification of GDSL-motif lipase/hydrolase (spot #3136) and calcineurin-like phosphoesterase (spot #1658) at 24 hai, suggests the potential involvement of these proteins in pathogen perception and signal transduction cascades. GDSL esterases/lipases are proteins with multifunctional properties, described as having a role in the regulation of plant development, morphogenesis, synthesis of secondary metabolites, and defense response (Chepyshko et al., 2012). In *Arabidopsis thaliana* a GDSL LIPASE1 protein seems to protect plants from *Alternaria brassicicola* attack in two distinct ways: by directly disrupting fungal spore integrity, and by activating defense signaling in the plants (Oh et al., 2005). In our study we have detected a decrease in the accumulation of the protein GDSL-motif lipase/hydrolase at 24 hai, in both infected tissues. According to Lee et al. (2009) such a decrease can be either a negative regulation of proteins to inhibit fungal infection/growth or, in addition, the effect of fungal interacting with the plant cell (by means of effector proteins) by suppressing the host immune system. On the other hand, the increased accumulation of calcineurin-like phosphoesterase (a calcium–dependent phosphatase) can be important in the regulation of various cellular processes with emphasizes in signal transduction as has already been shown (Kudla et al., 1999; Luan, 2003). It is known that upon perception of microbial signals, kinases and phosphatases target specific proteins, often modifying complex signaling cascades that allow for rapid defense responses (Delanois et al., 2014). The presence of phosphatases in the extracellular proteome of *Arabidopsis* infected with *Pseudomonas syringae* suggests that potential phosphorylation/dephosphorylation reversible regulation could occur in the apoplast (Kaffarnik et al., 2009). Moreover, Ndimba et al. (2003) have shown that chitosan treatment of *Arabidopsis* cell-suspentions induced phosphorylation of a receptor-like kinase, and other proteins like chitinases and glucanases (proteins that we have also found to be accumulated at 24 h, particularly in the R samples).

The increased accumulation of PR proteins (chitinases, osmotin and a cysteine-rich repeat secretory protein) in both infected tissues (more markedly in the R samples) also indicates the induction of the basal defense responses, possibly salicylic acid (SA) regulated. Molecular studies on *Coffea* spp.—*H. vastatrix* incompatible interaction did show the activation of genes (*ex.pr1b* and *gt*) known to be involved in the SA mediating signaling pathway around 21–24 hai (Diniz et al., 2012). Furthermore, SA quantification by HPLC/ESI-MS/MS showed an increase in this signaling compound at 24 hai in *Coffea* spp.—*H. vastatrix* incompatible interaction, suggesting again the involvement of an SA-dependent pathway in coffee resistance to CLR (Sá et al., 2014).

The accumulation of berberine bridge enzyme (a reticuline-like oxidase) in the R sample at 24 hai and a copper-zinc superoxide dismutase (SOD) in S sample at 48 hai, suggests that these “PR-like” proteins may co-regulate basal defenses. Extracellular oxidases have been suggested to catalyze the generation of reactive oxygen species (ROS), such as superoxide anions and hydrogen peroxide during the “oxidative burst” (Martinez et al., 1998; Mika et al., 2004). Indeed, previous cytochemical data in an incompatible *C. arabica—H. vastatrix* interaction, revealed hydrogen peroxide in the interface between the cuticle and the fungal pre-penetration structures at the infection sites (Silva et al., 2008). Furthermore, the increase in the activity of peroxidases, SOD and oxalate oxidases (germin–like proteins) have already been reported during the resistant response of coffee to CRL (Silva et al., 2006, 2008; Guerra-Guimarães et al., 2009a, 2013). The oxi-reductase activity observed during infection by pathogens indicates that plants were either initiating the production of ROS to fight directly the pathogen or responding to oxidative intermediates produced as a result of cell wall or membrane damage leading to cell death during HR response (Lee et al., 2009).

### Late/specific defense responses

Although the HR was already observed at 24 hai, it continued to increase with time and at 72 hai it was much higher in the R than in the S samples. Simultaneously, in the R sample the number of proteins changing in volume increased dramatically, suggesting their contribution to a second and stronger line of defense responses. On the contrary, in the S sample the fungus continued growing with no apparent inhibition, the HR stabilized and protein levels did not change much more than in the control. Most of the proteins that increased in the R sample at 72 hai have hydrolytic activity, being either involved in the cell wall metabolism (beta-xylosidase/alpha-arabinofuranosidases, chitinases and glucanase, pectin methylesterase, purple acid phosphatase, and reticuline oxidase) or in proteolysis (subtilases and serine carboxypeptidases).

It is known that plant glycohydrolases (GH) can play various important functions such as, cell wall expansion, modification during development, defense, and signaling. Since plant cell wall polysaccharides are very heterogeneous and complex polymers, GH activities must be very diverse (Jamet et al., 2008) and with our proteomic approach we identified in the apoplast a total of eight GHs superfamilies (3, 17, 18, 20, 31, 35, 38, 64). According to the carbohydrate-active enzymes database (CAZy; www.cazy.org) (Lombard et al., 2014), the GHs families GH3, GH31 and GH35 comprise enzymes that are mainly involved in the reorganization of cell wall carbohydrates. The other GHs families seem to be involved in glycoprotein post-translational modifications (PTMs), such as alpha-L-arabinofuranosidases (GH3), chitinases (GH18), beta-D-galactosidases (GH35), and alpha-D-mannosidases (GH38) (Jamet et al., 2008).

Alpha-L-arabinofuranosidases are particularly interesting since they accumulate only in the R sample at 72 hai. They are plant enzymes capable of releasing terminal arabinofuranosyl residues from cell wall matrix polymers (Saha, 2000), functioning as a candidate for a role in softening-related depolymerization of the cell wall during the HR response (Cantu et al., 2007). Several other apoplastic proteins identified, are also GHs, and appear to contribute to plant defense. Chitinases (GH18) and beta-1,3-glucanases (GH17) that are PR proteins possess antifungal activity limiting pathogen progression, and their expression is often triggered by pathogen infection (Silva et al., 2006; Guerra-Guimarães et al., 2009b). Leah et al. (1991) and Mauch et al. (1988) showed that the antifungal proprieties of plant chitinases are enhanced when beta-1,3-glucanases were added in combination with them. In transgenic tobacco plants, susceptibility to fungal attack decreased when chitinase and glucanase genes were both over-expressed (Zhu et al., 1994). Other PR-proteins such as PR-1 and PR-5 also increased in the resistant sample from 72 hai onwards.

Also relevant was the detection of pectin methylesterases (PMEs) and purple acid phosphatases (PAP) exclusively at 72 hai in the resistant sample. The activities of PMEs from both plants and pathogens and the degree and pattern of pectin methyl esterification are critical for the outcome of plant–pathogen infections. The cell walls containing highly methyl esterified pectin are somehow protected against the action of pathogens (Lionetti et al., 2012). Concerning the PAP, it was shown that a PAP5 is required for maintaining basal resistance against *Pseudomonas syringae* in *Arabidopsis*, suggesting a role for PAP5 in pathogen triggered immunity (Ravichandran et al., 2013).

Proteolytic enzymes that are thought to be involved in maturation of enzymes, signaling, protein turnover, and defense against pathogens (Jamet et al., 2008) were the proteins that mostly changed in abundance between the R and S samples, at 72 hai. They represent 36% of the total proteins identified and belong to 4 different superfamilies; subtilisin-like protease, serine carboxypeptidase, aspartic protease, and cysteine proteinase. Serine proteases (subtilases and serine carboxypeptidases) were the most relevant as they increased abundantly in the resistant sample, particularly at 72 hai. Several subtilases are specifically induced following pathogen infection and an *Arabidopsis* subtilase (SBT3.3) was very recently hypothesized to function as a receptor located in the plasma membrane that activates downstream immune signaling processes (Ramirez et al., 2013). When comparing grapevine genotypes resistant and susceptible to *Plasmopara viticola*, a subtilisin-like protein sharing sequence similarity with the tomato P69 (a PR protein specifically induced following pathogen infection) was shown to be constitutively expressed in the resistant genotype; and its expression was induced after pathogen infection (Vartapetian et al., 2011; Monteiro et al., 2013; Figueiredo et al., 2014).

In addition to the already referred functions of oxidases in the defense responses, it should be discussed the later increase in reticulin oxidase and germin-like proteins (oxalate oxidase-like) at 96 hai. These proteins can have a role in the oxidative cross-linking of cell wall proteins around the site of infection (Bradley et al., 1992; Silva et al., 2008). Crosslinks between phenolic compounds, the plant cell wall polysaccharides and proteins enhance the protection of the cell wall to digestion by microbial degrading enzymes and, thus, increase the global resistance to fungi (Bily et al., 2003). Deposition of chlorogenic acids and lignin has, indeed, been associated with the resistance of coffee to *H. vastatrix* (Silva et al., 2002, 2006; Leitão et al., 2011).

Overall, the protein changes occurring in the APF of coffee leaves upon *H. vastatrix* infection indicate that cell wall reorganization, accumulation of PR proteins and excretion of hydrolytic enzymes are likely to be important defense mechanisms of coffee. The use of antibodies produced against chitinase, pectin methylesterase, serine carboxypeptidase, reticuline oxidase, and subtilase showed an increased detection of these proteins in the incompatible interaction what strengthens their involvement in the resistant response of coffee against *H. vastatix*.

## Conclusions

Important constitutive defense proteins were revealed in the APF of *C. arabica* leaves. Upon infection by *H. vastatrix*, APF proteins were modulated establishing two distinct phases of defense responses, an initial/basal one (at 24–48 hai) and a late/specific one (at 72–96 hai). The number of proteins detected for the initial/basal phase is essentially half of the number of the proteins for the late/specific phase. When comparing the susceptible and resistant sample it was found that the increase in proteins was always greater in the resistant samples and more markedly in the late/specific phase. The resistant response involves the participation of several important groups of proteins, namely: GH of the cell wall, serine proteases (subtilases and carboxypeptidases) and PR proteins. The GHs confer great plasticity to cell wall polysaccharides, the proteases (together with phosphatases) lead to a complex regulation of cell wall proteins through PTMs and PR proteins are directly involved in antifungal activity. These results suggest that some glycohydrolases, proteases, and PR-proteins are putative candidates for resistant markers of coffee to CLR. The production of antibodies against chitinase, pectin methylesterase, serine carboxypeptidase, reticuline oxidase, and subtilase enabled the validation of the importance of these proteins in the coffee resistance response by immunodetection assay. Reliability of these putative resistant markers will be subsequently tested in several well-known coffee cultivars with commercial value. The genes corresponding to the protein biomarkers can be integrated in marker-assisted breeding programs aiming to assist in the selection of appropriate coffee genotypes with resistance to *H. vastatrix*.

### Conflict of interest statement

The authors declare that the research was conducted in the absence of any commercial or financial relationships that could be construed as a potential conflict of interest.
